# Recent advances in functional bowel disorders

**Published:** 2016

**Authors:** Dominic King, David Al Dulaimi

**Affiliations:** *Department of Gastroenterology, Alexandra Hospital, Redditch, UK*

*Shino Shimura et al.* ***Small Intestinal Bacterial Overgrowth in Patients with Refractory Functional Gastrointestinal Disorders*** *. J Neurogastroenterol Motil 2016;22:60-68.*

Functional gastrointestinal disorders (FGID) are common; however, despite their prevalence the underlying pathophysiology is not well understood. The interest in the microbiome of the gut, and the differences in fermentation seen in patients with irritable bowel syndrome (IBS)and functional dyspepsia (FD) have led some researchers to suggest that small intestinal bacterial overgrowth (SIBO) may play a role. 

Standard therapy for FGDI in the form of antidepressants, prokinetics and antacids is likely to have a limited role in symptom of SIBO sufferers. In this study, the authors examined the frequency of SIBO in patients with refractory FGDI using Glucose Hydrogen Breath Testing (GHBT). The authors defined FGDI as failure of afore mentioned pharmacological therapies for 4 weeks or more, fulfilling Rome III criteria, without alternative organic cause to explain symptoms and effort was taken to exclude confounding variables.

Enrolled patients testing positive for SIBO (2/38) were treated with Levofloxacin for 7 days. 1 patient had IBS and one had FD, both were female and negative for *Helicobacter Pylori. *Following antibiotic treatment GHBT was redone and both participants showed normal patterns.

Patients were assessed before and after treatment using the Izumo Scale (a self-assessment of abdominal symptoms effect on quality of life). Post treatment assessment showed a reduction in symptoms of fullness though little other changes in symptoms.

The authors comment that the Japanese study cohort may have lower SIBO than the literature has suggested in other populations (ranging from 2-84% in IBS sufferers), and likely reflects variations in SIBO diagnostic tests, diet and geographic variations in small intestinal microbiome. 

The authors concede that their cohort is unlikely to be generalizable due to the low rates of SIBO in Japan that their cohort was sourced from a tertiary centre. However they stress that SIBO is an important differential in hard to treat patients with IBS or FD.

*Per G. Farup et al.* ***Faecal short-chain fatty acids a diagnostic biomarker for irritable bowel syndrome?*** *BMC Gastroenterology (2016) 16:51*

The diagnosis of Irritable Bowel Syndrome (IBS) is based on the Rome consensus and is a diagnosis of exclusion. Frequently, multiple investigations are conducted before a diagnosis has been made. A reliable biomarker of IBS has thus long been sought.

Short Chain Fatty-Acids (SCFA) are produced by gut microbiota from non-digested food in the gut. IBS has been associated with the gut flora, and interactions with the host’s immune system via SCFA may be a causative link.

In Farup et al’s case-control study, 25 patients with IBS (according to Rome III criteria) and 25 without were recruited to examine the potential for a biomarker of IBS. Faecal samples were analysed from participants using gas chromatography and SCFA type (Acetic acid, Propionic acid, Butyric acid, Iso-Butyric acid, Valeric acid, Iso-Valeric acid) and proportion were characterised. 

They found statistically higher levels of Butyric acid in the control group (*p* = 0.003) and a non-significant, though trend towards higher levels of Propionic acid in the IBS group (*p *= 0.07). The Pro-But (mmol/L) difference was highly significant (*p* = <0.001) with an area under the ROC curve of 0.89 (95 % CI 0.80: 0.98), p < 0.001. The authors gave a value of <0.13 as able to exclude IBS with a sensitivity of 100% and a value of >0.46 as having a 100% specificity for IBS. 

Farup et al stress the need for a biomarker for daily use and suggest SCFA may be a valuable tool in the diagnosis/exclusion of IBS. They accept that the lack of a gold standard for IBS diagnosis and comparison with consensus Rome criteria is not ideal. There is a lack of consistency both in this study and others regarding the amount of SCFA in patients and the authors suggest this may be due to opposing effects of different SCFA, and that the *relative* amounts may be pivotal.

This study’s findings show promise, though the authors admit a small study with participants taken from an earlier study looking at depression vs unspecified neurological symptoms. They stress that statistical analysis found no confounders however. Further, new studies which not only look at IBS vs healthy volunteers but also organic disease will better characterise faecal SCFA as a potential non-invasive, easy to use biomarker test.

*Ramin Niknam et al.* ***Self-medication of irritable bowel syndrome and dyspepsia: How appropriate is it?*** *J Res Pharm Pract. 2016 Apr-Jun; 5(2): 121–125.*

The inappropriate use of medications is a global problem, and Iranian medication consumption appears to be higher than in many other countries. Self-medication is common for gastrointestinal symptoms and may be inappropriate for the symptoms they are intended to treat.  

Niknam et al’s study looked at the use of self-medication in irritable bowel syndrome (IBS) and functional dyspepsia (FD) and whether the medication chosen was suitable.

337 patients with IBS and 410 with FD aged over 12 were included in this prospective cross-sectional study based on Rome III criteria diagnosed by a gastroenterology specialist. 78% were of *Fars *ethnicity and the cohort’s average age was 40. *UpToDate *guidance on IBS management was used as a standard against which appropriateness was judged although no standard for herbal medications could be found.

H2 antagonists were the most commonly used medication for both IBS and FD, with 785 of all patients using some form of non-prescribed medication for their GI symptoms. 28% of participants were deemed to have used inappropriate medications for their GI symptoms. Those with IBS-constipation predominant symptoms were the group with the most inappropriate medication use (80%). No statistical significance was shown between appropriate selection of medications and the recorded demographics of age, level of education, or marital status.

The authors speculate that the high use of antacids in IBS may represent frustration with standard therapies and other concurrent GI symptoms. Of note the high use of antacids for IBS was observed in other studies outside of Iran. The low use of antidepressants for IBS in the study may be due to a lack of knowledge of these medications in primary care. High cost of healthcare and ease of access to non-prescription medication was cited both in Iran and other developing countries as the main reason for self-medication.

Finally the authors propose limitation to non-prescription medications, increased ease of access to physicians and education programmes to facilitate more appropriate GI symptom management. 

*Louise Maagaard et al.* ***Follow-up of patients with functional bowel symptoms treated with a low FODMAP diet****. World J Gastroenterol. 2016 Apr 21;22(15):4009-19.*

Irritable Bowel Syndrome (IBS) is common and IBS-like symptoms are frequently seen in patients with Inflammatory Bowel Disease (IBD). IBS has diverse characteristics and as such managing it can be challenging. 

Exclusion of FODMAPs (fermentable oligo-, di-, and monosaccharides and polyols) from the diet has found to be of benefit to IBS sufferers with good response rates. FODMAPS are not well absorbed in the small bowel and their passage to the colon exerts and osmotic effect and increased gas production. The exclusion of these fermentable products from the diet is complex for patients. This retrospective study, using questionnaires, sought to examine the long-term impact and adherence to a low FODMAP diet in participants with IBS and IBS with IBD.

Respondents were given expert advice and education on low FODMAP diets and IBS and encouraged to persist with the diet for 6-8weeks following which their disease course would be assessed. Questionnaires with expert consensus thresholds for adherence to diet and satisfaction with diet were provided to participants and a further questionnaire reviewed the course of IBS symptoms prior to and following diet implementation. IBD patients were provided with an IBD specific questionnaire at follow up with reviewed quality of life in IBD.

A total of 180 patients which fulfilled inclusion criteria responded with 131 in the IBS group and 49 in the IBD group. 84% of patients reported some or total improvement in symptoms with the diet. The IBD group reported the most full effectiveness of the diet (42% *vs* 29%, *P =* 0.08). IBD patients at follow up had fewer symptoms than IBS patients. Bloating and pain symptoms had the greatest resolution while following the diet. The diet also saw significant improvements in stool consistency.

Adherence was only seen in a third of patients and although only half of patients were on the diet by follow up, most were satisfied with the treatment.

The authors comment that this is the first review of the long term effects of a low FODMAP diet and although caution is advised due to the change in gut microflora and the limitations of a retrospective study and poor response rate (48% non-responders), they support the findings and advise larger and longer term studies of the use and safety of these diets.

*Saeedeh Zomorrodi et al.* ***Long Term Effects of Mindfulness on Quality of life in Irritable Bowel Syndrome*** *Iran J Psychiatry 2015; 10:2: 100-105.*

Irritable Bowel Syndrome (IBS) is the most common of the functional bowel disorders and is both challenging and expensive to manage. Psychological symptoms are reported in the majority of IBS sufferers and these aspects of the condition require more investigation. Health-related quality of life in IBS can be used as a surrogate for disease course, partly because of the unsatisfactory diagnostic criteria and partly because of the significant impact on quality of life IBS sufferers report. 

Cognitive Behavioural Therapy (CBT) has been found to be of most success in treating IBS and is based on the premise that dysfunctional thinking leads to disordered cognitive processes and impacts on brain-bowel neural connections. However, the literature reports no long-term maintained effects and others have found no benefit whatsoever for IBS sufferers. 

Mindfulness-based Therapy (MFT) is a way of providing psychological well-being by overcoming automatic negative thoughts and some suggest it has long lasting effects in IBS. Zomorrodi et al’s experimental study looked at an intervention and control group (12 participants each). Those in the control group received only medical therapy and the intervention group included medical therapy and MFT 8 week, 2-hourly sessions focused on stress reduction. IBS-QOL-34 (Quality of Life) questionnaire by Patrick and Drassman was conducted before and after the intervention.

The study included 13 men and 11 women with a mean age of 34. It found a statistically significant improvement in the intervention (using one-way co-variance analysis) showing evidence of a long-term benefit.

The study concedes limitations due to the lack of a sham intervention in the control group and a small sample size, but suggests that mindfulness techniques enable patients to re-establish positive cognitive practices allowing negative elements to be viewed as only transient and not embedded.

*Andrés Acosta et al.* ***Effects of Rifaximin on Transit, Permeability, Fecal Microbiome, and Organic Acid Excretion in Irritable Bowel Syndrome.*** *Clinical and Translational Gastroenterology* *(2016).*

It has been suggested Irritable Bowel Syndrome (IBS) is the result of psychological, mucosal sensitivity, motility and immunological disordering. Previous infections appear to increase the risk of developing IBS and the suggestion that changes to the gut microbiome and subsequent effects on colonic permeability, Short Chain Fatty Acids (SCFAs) and bile acids may also contribute to the IBS are explored in this study. 

The non-absorbable antibiotic, Rifaxamin, has been previously shown to improve symptoms in IBS and is licensed for use in IBS-D. The double-blinded, placebo controlled trial by Acosta et al. took 24 participants with non-constipation IBS (IBS-D or IBS-M) and randomised them to placebo group or treatment with Rifaxamin 550mg three times a day for 14 days. Patients with confirmed previous Small Intestinal Bacterial Overgrowth (SIBO) were excluded but screening tests for SIBO was not undertaken (the authors feeling that the tests available lack sensitivity and gold-standard duodenal aspirates being too complex).

Stool frequency and consistency were assessed using patient questionnaires and patients kept a diary through the treatment phase. Bowel transit using scintigraphy and bowel wall permeability were calculated using assessment of urine excretion of lactulose and mannitol after oral administration. The microbiome of the gut flora was reviewed using bacterial 16S rRNA gene PCR amplification, and bile acid and faecal SCFA were measured both pre-treatment and in the final 48hours of the treatment phase.

The 2 groups were well matched although the intervention arm had higher bile acid excretion at baseline. 

There was found to be no significant effect on stool consistency or bowel permeability with Rifaximin. Increased ascending colon emptying and minimal, though significant increase in whole colon transit speed at 48 hours was demonstrated. Rifaximin had no statistical effect on Bile acid or faecal SCFA excretion. Although there were changes in the makeup of the microbiome in the treatment arm, these were too small to have statistical relevance. 

The authors conclude that although previous studies have shown improved IBS symptoms with Rifaximin, the mechanism for this requires further study.

